# Simultaneous Hierarchical Bayesian Parameter Estimation for Reinforcement Learning and Drift Diffusion Models: a Tutorial and Links to Neural Data

**DOI:** 10.1007/s42113-020-00084-w

**Published:** 2020-05-26

**Authors:** Mads L. Pedersen, Michael J. Frank

**Affiliations:** 1 Cognitive, Linguistic & Psychological Sciences, Brown University, Providence, USA; 2 Robert J. & Nancy D. Carney Institute for Brain Science, Brown University, Providence, USA; 3 Department of Psychology, University of Oslo, Oslo, Norway

**Keywords:** Computational modeling, Reinforcement learning, Decision making, Toolbox, Drift diffusion model, Reinforcement learning drift diffusion model

## Abstract

Cognitive models have been instrumental for generating insights into the brain processes underlying learning and decision making. In reinforcement learning it has recently been shown that not only choice proportions but also their latency distributions can be well captured when the choice function is replaced with a sequential sampling model such as the drift diffusion model. Hierarchical Bayesian parameter estimation further enhances the identifiability of distinct learning and choice parameters. One caveat is that these models can be time-consuming to build, sample from, and validate, especially when models include links between neural activations and model parameters. Here we describe a novel extension to the widely used hierarchical drift diffusion model (HDDM) toolbox, which facilitates flexible construction, estimation, and evaluation of the reinforcement learning drift diffusion model (RLDDM) using hierarchical Bayesian methods. We describe the types of experiments most applicable to the model and provide a tutorial to illustrate how to perform quantitative data analysis and model evaluation. Parameter recovery confirmed that the method can reliably estimate parameters with varying numbers of synthetic subjects and trials. We also show that the simultaneous estimation of learning and choice parameters can improve the sensitivity to detect brain–behavioral relationships, including the impact of learned values and fronto-basal ganglia activity patterns on dynamic decision parameters.

## Introduction

Computational models of learning and decision making have contributed substantially to a large body of research aimed at understanding the link between neural systems and behavioral output. For example, the discovery that phasic dopamine in the midbrain signals a reward prediction error in reinforcement learning (RL; Montague, Dayan & Sejnowski, 1996) has been extended to explore their downstream effects in striatum to accommodate various findings in instrumental choice (Collins and Frank 2014). Similarly, sequential sampling models of decision making have been widely used to interpret the link between neural patterns of activity rising to a threshold and their impact on choice (Ding and Gold 2010; Doi et al. 2019; Gold & Shadlen, 2007; O’Connell et al. 2012; Shadlen & Newsome, 2001, Smith and Ratcliff 2004).

In part motivated by theoretical considerations linking RL to sequential sampling (Bogacz and Larsen 2011; Dayan and Daw 2008), empirical and methodological studies have combined these two traditionally separate models (Fontanesi et al. 2019; Frank et al. 2015; McDougle and Collins 2019; Pedersen et al. 2017; Sewell et al. 2019; see Miletić et al. 2020, for review). The aim of these models is to capture not only the evolution of choice proportions in reinforcement learning but also the speed (and distribution thereof) with which these decisions are made. These approaches assume that choices between reinforced options can be described as a sequential sampling process where evidence is accumulated until reaching a threshold to respond. Incorporating response times affords a richer interpretation of the mechanisms underlying choice in RL (Pedersen et al. 2017; Frank et al. 2015), can better describe observed behavioral patterns such as magnitude effects (Fontanesi et al. 2019), and even improves identifiability of learning parameters (Ballard and McClure 2019; Shahar et al. 2019).

A current limitation with these models, and cognitive models in general, is that they are time-consuming and complicated to apply to new datasets, as they usually require writing custom scripts that are inflexible to model variations. This issue is particularly salient when the goal is to estimate the impact of neural activation onto behavioral model parameters. Here we present a toolbox developed for the reinforcement learning drift diffusion model (RLDDM; Pedersen et al. 2017). The RLDDM builds on the drift diffusion model (DDM; Ratcliff 1978), a sequential sampling model of two-alternative decision making, which, when applied to value-based decisions, assumes that the scaled difference in expected values between options is accumulated during choice. The RLDDM further estimates the learning rate at which these expected values evolve with probabilistic outcomes across learning, based on RL principles. We describe a novel RLDDM module as an extension to the open-source HDDM python package (Wiecki et al. 2013) that allow users to easily model data with the RLDDM and estimate the correlation of trial-by-trial neural activation and learning and decision parameters.

This tutorial begins by first providing a brief introduction to the modeling formalism (see Pedersen et al. (2017) for more details). We proceed with a description of the types of tasks applicable to the model, followed by the steps needed to fit a basic model, and various methods for performing model validation, including posterior predictive checks. We then present an RLDDM regression module that can be used to include neural (or other trial-by-trial) regressors to estimate how they impact model parameters. We perform a parameter recovery study showing how recoverability of parameters varies dependent of the number of trials and number of subjects in the data, serving as a guide in choosing the minimum sample size and trials required to reliably estimate parameters. Finally, we show that the RLDDM replicates previous findings linking neural activity in frontal cortex and basal ganglia on dynamic decision parameters (Frank et al. 2015) but also improves the sensitivity to detect such effects.

## Methods

### The Reinforcement Learning Drift Diffusion Model

Reinforcement learning (RL) models describe the process of learning the value of stimuli and actions. The two main components of an instrumental RL model is a mechanism for describing how reward feedback is used to update value expectations and a mechanism describing how an agent use these expectations to make a choice. A common way to formalize the learning process is the delta learning rule (Rescorla and Wagner 1972; Watkins and Dayan 1992), which prescribes the updating of the expected value of option *i* as

(1) $$Q_{o,i}=Q_{o,i-1}+\alpha \left({\text{Reward}}_{o,i-1}-Q_{o,i-1}\right)$$

where the expected (*Q*) value for option *o* on trial *i* is updated in proportion to the reward prediction error, i.e., the difference between observed and predicted reward, scaled by the learning rate *α*.

The choice rule for selecting among reinforced options is commonly described by the softmax logistic function (Luce 1959):

(2) $$p_{o,i}=\frac{e^{\left(\beta \times Q_{o,i}\right)}}{\sum_{j=1}^{n}e^{\left(\beta \times Q_{j,i}\right)}},$$

where the inverse temperature parameter *β* scales the probability of choosing option *o* as a function of how much larger its expected value is compared to other options *n*. Increased inverse temperature is related to stronger tendencies to exploit current knowledge, leading to more deterministic choices, whereas lower values are associated with exploration of options with lower, but potentially more uncertain, expected reward.

The softmax logistic function can accurately capture trial-by-trial choices between reinforced options (e.g.. Daw et al. 2006), but it does not describe the cognitive process of choice and cannot differentiate between choices that are fast and accurate from those that are slow and conservative. Conversely, sequential sampling models capture the dynamics of the choice process and their impact on response time distributions. The aforementioned combined RL and DDM models (Fontanesi et al. 2019; McDougle and Collins 2019; Pedersen et al. 2017) attempt to describe the cognitive mechanisms of choice in reinforcement learning by replacing the softmax choice rule with a sequential sampling model in which evidence about choice values is continuously sampled until reaching a predetermined decision threshold in favor of an alternative.

Here we focus on the RLDDM described in Pedersen et al. (2017), which uses the DDM ( Ratcliff 1978) to capture choice and response time. The DDM models two-alternative forced choice decision making with the drift rate (parameter *v*), which describes the rate of (noisy) evidence accumulation, where the amount of evidence required to commit to a decision is modeled with a decision threshold (parameter *a*). The non-decision time parameter *t* captures time spent on sensory perception, motor preparation, and motor output, and the starting point parameter *z* captures potential biases in the initial decision variable toward one or the other boundary. Finally, the so-called “full” DDM (Ratcliff & Rouder, 1998) includes parameters capturing between-trial variability of evidence accumulation, non-decision time, and starting point. Importantly, these parameters have separable effects on choice and response time, allowing parameter values to be disentangled. For example, larger drift rates produce faster and more accurate decision making, whereas larger thresholds produce slower and more accurate decision making. Further, the process of evidence accumulation is assumed to be noisy, which captures how seemingly identical decision making alternatives can lead to different decisions and response times. The DDM is most commonly used to explain the cognitive processes of perceptual decision making but has more recently been shown to also provide good fits to value-based decision making (e.g., Basten et al. 2010; Cavanagh et al. 2014; Krajbich et al. 2010; Ratcliff and Frank 2012; Westbrook et al. 2020, in which the preference of options is assumed to remain stable across decisions. RLDDMs further assume that drift rates depend on the trial-by-trial difference in expected rewards (Fontanesi et al. 2019; Frank et al. 2015; McDougle and Collins 2019; Pedersen et al. 2017; Sewell et al. 2019). Replacing the softmax with the DDM for reinforcement learning can be seen as an extension of the softmax choice function to account for the latency of the choice, as the likelihood of choice between two options in the softmax is equivalent to a DDM that ignores RT (see, e.g., Miletić et al. 2020).

The RLDDM described here replaces the softmax choice rule with the DDM by assuming that the rate of evidence accumulation in reinforcement-based decision making can be described by the scaled difference between the expected value of reinforced options:

(3) $$v_{i}=\left(Q_{\text{upper},i}-Q_{\text{lower},i}\right)*v,$$

where *Q*_upper_ (*Q*_lower_) is the expected value of the option associated with the upper (lower) decision threshold and *v* is a free parameter that describes the agents’ degree of exploration/exploitation, similar to the inverse temperature *β* parameter in the softmax choice rule described above. The RLDDM uses the same probability distribution as the normal DDM, namely, the Wiener first passage time (wfpt) distribution:

(4) $$rt_{o,i}∼\text{wfpt}\left(a,t,z,v_{i}\right),$$

where *rt*_*o*_, _i_ is the response time for choosing option *o* on trial *i* and the wfpt returns the probability to choose *o* with response time rt. Note that the RLDDM models described here refer to drift rate *v_i_* as the trial-by-trial product of the difference in estimated values (*Q*_upper, *i*_ – *Q*_lower,*i*_) and the scaling parameter *v*.

### Extensions to the HDDM Toolbox with the RLDDM Module

**Fig. 1:**
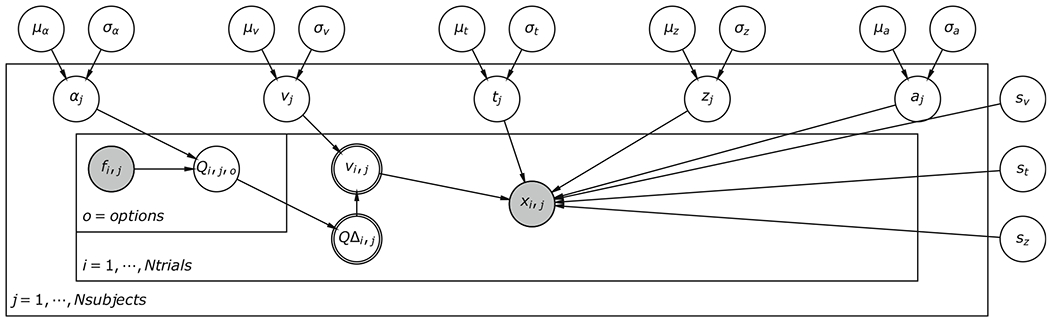
Graphical model of the reinforcement learning drift diffusion model (RLDDM). Round nodes are random variables; nodes with double borders are deterministic variables. Shaded nodes represent observed data. Subject parameters are drawn from group mean *μ* and variance *σ* parameters. Trial-by-trial drift rates (*v_i,j_*) depend on the difference in expected values QΔ scaled by the scaling parameter *v*, where *Q* values are updated dependent on the feedback *f* and learning rate *α*. *a* = decision threshold, *v* = drift rate scaling parameter, *t* = non-decision time, *z* = starting point *α* = learning rate, *f* = feedback, *Q* = expected value, *x* = choice and response time, *s_v_* = between-trial variability in drift rate, *s_t_* = between-trial variability in non-decision time, and *s_z_* = between-trial variability in starting point

To allow users to flexibly create RLDDM models (e.g., to estimate how separable parameters vary with condition or neural activity) we built extensions to the HDDM toolbox (Wiecki et al. 2013). HDDM is an open-soruce python software package that uses Markov chain Monte Carlo slice sampling via PyMC (Patil et al. 2010) to approximate the posterior distribution of group and individual parameters using a hierarchical Bayesian framework. Advantages of Bayesian hierarchical models include directly describing uncertainty of parameter estimates through the width of the posterior distribution (Kruschke 2010), and because group and subject parameters mutually inform each other, they require fewer trials (compared to non-hierarchical Bayesian methods, maximum likelihood estimation, and χ^2^ quantile methods) to accurately recover parameter values (Wiecki et al. 2013). For similar reasons they also facilitate the robust identification of links between noisy neural signals and variability in model parameters (Wiecki et al. 2013). HDDM uses the approximation described by Navarro and Fuss (2009) to calculate the wfpt probability density. The RLDDM module in HDDM can be represented with the graphical model in Fig. 1 and uses the following prior distributions for parameters:

(5) $$\mu_{a}∼G\left(1.5,0.75\right)\;\sigma_{a}∼HN\left(0.1\right)\;a_{j}∼G\left(\mu_{a},\sigma_{a}^{2}\right)\;\mu_{v}∼N\left(2,3\right)\;\sigma_{v}∼HN\left(2\right)\;v_{j}∼N\left(\mu_{v},\sigma_{a}^{2}\right)\;\mu_{z}∼N\left(0.5,0.5\right)\;\sigma_{z}∼HN\left(0.5\right)\;z_{j}∼\text{invlogit}\left(N\left(\mu_{z},\sigma_{z}^{2}\right)\right)\;\mu_{\alpha }∼N\left(0,3\right)\;\sigma_{\alpha }∼HN\left(2\right)\;\alpha_{j}∼\text{invlogit}\left(N\left(\mu_{\alpha },\sigma_{\alpha }^{2}\right)\right)\;\mu_{t}∼G\left(0.4,0.2\right)\;\sigma_{t}∼HN\left(1\right)\;t_{j}∼N\left(\mu_{t},\sigma_{t}^{2}\right)\;s_{v}∼HN\left(2\right)\;s_{t}∼HN\left(0.3\right)\;s_{z}∼B\left(1,3\right)$$

where N represents a normal distribution parameterized by mean and standard deviation; HN represents a positive-only, half-normal distribution parameterized by standard-deviation; G represents a Gamma distribution parameterized by mean and rate; and G represents a Beta distribution parameterized by alpha and beta. An inverse logit (invlogit) transformation is used to transform normally distributed parameters to the range 0 and 1. Note that HDDM uses the relative parameterization of the DDM where the starting point parameter represents the relative starting point between decision thresholds and is bound to be between 0 and 1. The priors used for DDM-parameters are informative priors selected from a meta-analysis of prior DDM studies (Wiecki et al. 2013), whereas the learning rate parameter is non-informative using a wide normal distribution that when transformed is centered at 0.5. Although parameter recovery studies show good performance for recovering learning rate parameters closer to 0, future versions of the RLDDM models could further improve by using estimated values from RLDDM studies to inform the empirical prior for learning rate parameters. The “full” DDM with between-trial variability parameters *s_v_*, *s_t_* and *s*_*z*_ can also be added (see online tutorial) and are by default set to be group-only parameters because they are commonly difficult to recover at the individual level (Boehm et al. 2018).

### Tutorial

Here we describe some of the functionality of the RLDDM modules in HDDM. This tutorial shows how to use the RLDDM modules to simultaneously estimate reinforcement learning parameters and decision parameters within a frilly hierarchical Bayesian estimation framework, including steps for sampling, assessing convergence, model fit, parameter recovery, and posterior predictive checks (model validation). More details, including a more comprehensive tutorial with instruction on installing the package, can be found online at http://ski.clps.brown.edu/hddm_docs/.

### Overview of Models

The HDDM software has two RLDDM modules, and an additional option to include only classical RL without RTs using the softmax function to model choices (but still in a hierarchical Bayesian framework). The HDDMrl module is built to estimate group and subject parameters with hierarchical Bayesian estimation. Models can be set up to assume that subjects utilize the same learning and decision parameter values across conditions. Alternatively, it can be set up as a between-subject model assuming one or more parameters vary as a function of discrete conditions. The HDDMrl cannot be used to estimate the impact of continuous regressors within a condition or to estimate within-subject effects. For this purpose, users can utilize the HDDMrlRegressor module. Lastly, the Hrl module has the same functions as the HDDMrl module but captures choosing using the softmax choice function instead of the DDM and therefore does not require that the data include reaction times.

### Data Requirements

RLDDM is designed to analyze data from instrumental learning tasks where participants on a given trial choose between two response options, e.g., selecting between two stimuli or selecting the correct response for a single stimulus. The distribution of feedback can take any form, e.g., binary probabilistic outcomes or randomly distributed outcomes. RLDDM (or HDDM in general) currently cannot analyze tasks with more than two response options, but we plan to extend this capability in the near future. The models are also not designed to keep track of more than two expected values within a condition, as for example in an experiment where subjects in one trial choose between options A and B and in the next choose between options B and C.

### Structuring Data

The models take a pandas dataframe (https://pandas.pydata.org/) as input, where each row represents a trial, in ascending order. The following columns are required:

**rt**. Response time in seconds.

**response.** The chosen action (or stimulus in the case of a stimulus selection task) where one alternative is associated with the upper bound (response = 1) and the other with the lower bound (response = 0).

**split_by**. Defines task conditions (trial-types). The trials need to be split by condition to ensure proper updating of expected values only for the corresponding trial type.

**subj_idx**. Numerical value identifying the subject on the current trial.

**feedback**. The numerical feedback received for the chosen option on the current trial.

**q_init.** This value is used to initialize expected rewards. It can be any value, but an unbiased initial value should be somewhere between the minimum and maximum reward values (e.g., 0.5 for tasks with rewards of 0 and 1).

### Running Basic Model

To illustrate how to run the model we will use example data from the learning phase of the probabilistic selection task (PST) (e.g., Frank et al. 2004). During the learning phase of the PST subjects choose between two stimuli presented as Hiragana-letters (here represented as letters from the Latin alphabet). There are three conditions with different probabilities of receiving reward (feedback = 1) and non-reward (feedback = 0). In the AB condition A is rewarded with 80% probability and B with 20%. In the CD condition C is rewarded with 70% probability and D with 30%, while in the EF condition E is rewarded with a 60% probability and F with 40%. The dataset is included in the installation of HDDM. Once the data are uploaded a model can be created and ran with the following commands:

**Table:** 

m = hddm.HDDMrl (data)
m.sample (1500, burn=500)

The first call creates a model from the class HDDMrl with the data as input. The second call runs the specified model by sampling from the posterior distribution, with inputs defining the number of samples (here 1500) and the number of initial samples that should be discarded as bum-in, during which the MCMC sampler identifies the region of most likely values.

A summary of the posterior distribution of the parameters can be displayed by calling the function print_stats():

**Table:** 

m.print_stats ()							
	mean	std	2.5q	25q	50q	75q	97.5q
a	0.857	0.076	0.712	0.804	0.857	0.905	1.02
a_std	0.401	0.060	0.300	0.359	0.395	0.434	0.541
a_subj.1	1.036	0.089	0.86	0.97	2.03	1.09	1.228
a_subj.2	1.102	0.052	1.006	1.065	2.102	1.137	1.212
v	3.328	0.572	2.310	2.937	3.318	3.697	4.525
alpha	−2.32	0.312	−2.96	−2.53	−2.32	−2.11	−1.73
alpha_std	1.385	0.273	0.928	1.191	1.363	1.552	2.002

The columns in the table represent the mean, standard deviation, and quantiles of the approximated posterior distribution of each parameter. The default HDDMrl model estimates group and subject parameters for the following latent variables:

*a* = decision threshold
*v* = scaling parameter onto drift rate
*t* = non-decision time (not shown here)
alpha = learning rate.

Note that the estimated learning rate is not bound between 0 and 1, because it was transformed to improve sampling. To transform alpha back to the range 0–1 one can apply the inverse logit: *e*^alpha^/(1 + *e*^alpha^), which in this case gives a mean of 0.09 for the posterior distribution of the learning rate.

### Assessing Results

**Fig. 2:**
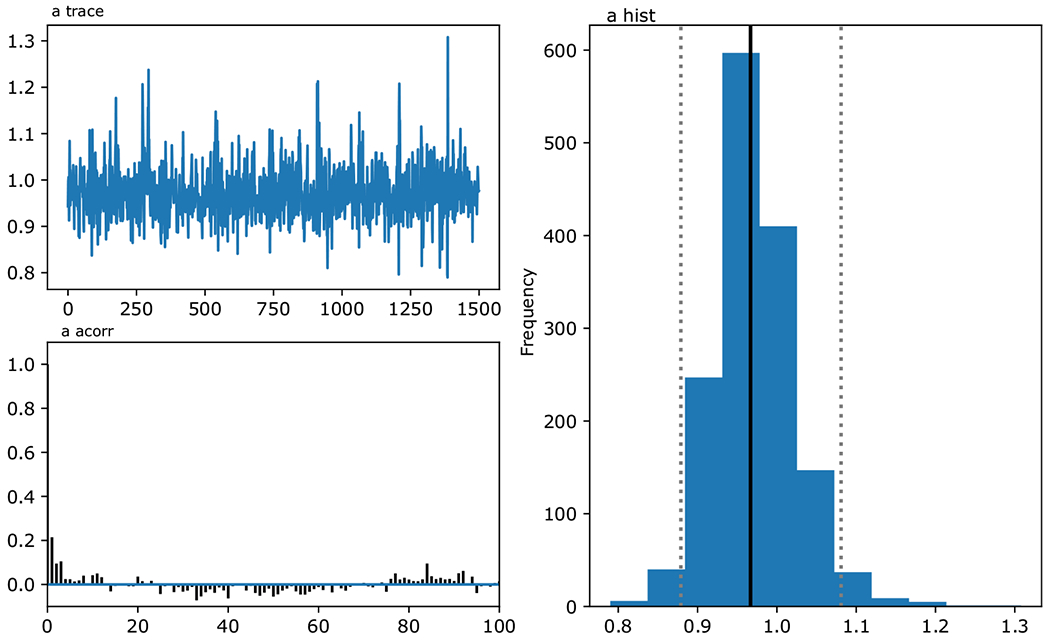
Plot of posterior distributions here exemplified by the group parameter for decision threshold *a*. The top left plot shows the sampled value for the parameter across samples, the bottom left shows the autocorrelation of samples, and the right figure shows a histogram of the approximated posterior distribution

HDDM includes functions to assess the model output by investigating the posterior distribution. Here we use model.plot_posteriors(), which gives information on the values of the samples, their autocorrelation (indicating the unique information in each sample), and the histogram of the posterior distribution for parameters (Fig. 2). As for any MCMC application, the mixing of the chain should ideally look like a “furry caterpillar” (stationary mean with some variance around it), the autocorrelation should quickly go to zero, and the histogram should look normally (or unimodally) distributed.

The Gelman–Rubin statistic (Gelman and Rubin 1992) is a metric measuring the degree of variation between and within chains indicating whether the chains in the model have converged. Values close to 1 indicate convergence and that there is small variation between chains, i.e., that the asymptotic distribution is similar for each chain. A common heuristic is to assume convergence if all values are below 1.1. As shown in the code below, to get a Gelman–Rubin statistic, users need to ran the model multiple times, combine the models, and then apply the gelman_rubin function. Increasing number of samples can help if there are problems with convergence, as the resulting samples should be a closer approximation to the hue posterior distribution.

models = []
for i in range(3):

**Table:** 

models = []
for i in range(3):
m = hddm.HDDMrl (data=data)
m.sample (1500, burn=500)
models.append (m)

gelman_rubin(models)

{‘a’: 1.000,
‘a_std’: 1.000,
‘a_subj.1’: 1.000,
‘a_subj.2’: 1.000,
‘v’: 1.001,
‘alpha’: 1.003,
‘alpha_std’: 1.001}

### Posterior Predictive Checks

**Fig. 3:**
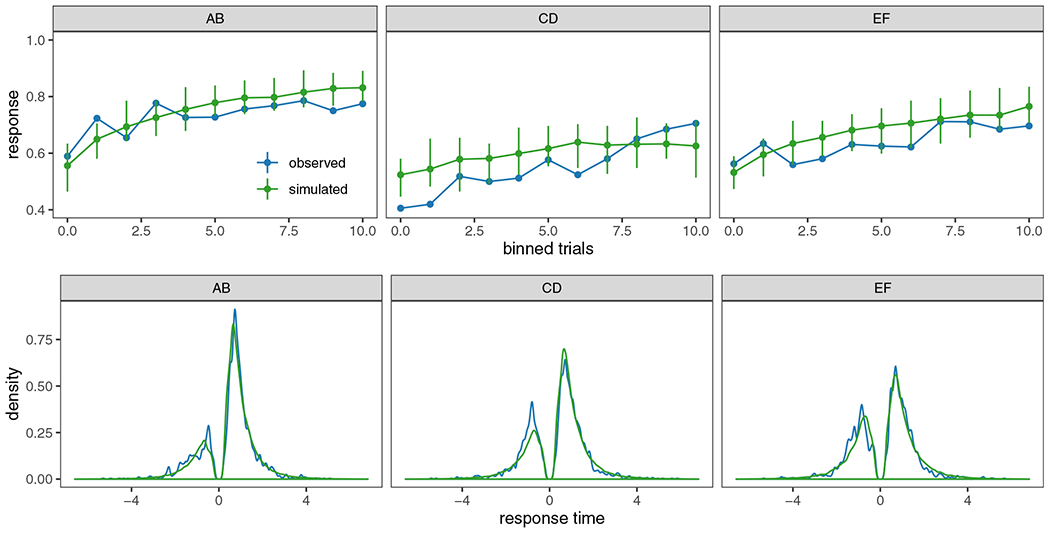
Posterior predictive checks. Top, observed (blue) and predicted (green) choice proportion of best option (response = 1) through learning and across conditions with reward probabilities of best and worst option of 0.8 and 0.2 (AB), 0.7 and 0.3 (CD), and 0.6 and 0.4 (EF), respectively. Trials were binned to create smoother curves, where each bin represents 4 trials; i.e., participants performed 40 trials in each condition. In general, the model generates data that closely follows the observed behavior, with the exception of overpredicting performance early in the intermediately difficult condition (condition CD). Uncertainty in the generated data is captured by the 90% highest density interval of the means across simulated datasets. Bottom, density plots of observed (blue) and predicted (green) response time across conditions. RTs for lower boundary choices (i.e., worst option choices) are plotted as negative (0-RT) to facilitate visualization of proportions and latency distributions for upper and lower bound responses. Here, the model generally captures the response time distribution. Note that for the CD case, the model generally captures the shapes of the distributions but again shows that the proportion of choices of the suboptirnal option (D) is underpredicted

It is not sufficient to fit a model and to only check for convergence before assessing its parameters. An important model validation step is to assess whether the model with fit parameters is sufficient to reproduce key features of the observed data. The Bayesian approach to this step is called a posterior predictive check, because it simulates data by sampling from the full posterior distribution of parameter estimates. One can then check if the observed patterns of data are within the predicted range taking into account the uncertainty of the parameters. The toolbox includes functions to generate data. For the sake of brevity, we refer the reader to the online tutorial (http://ski.clps.brown.edu/hddm_docs/) to see the code for generating data with estimated parameters. Here we show how well the model can recreate observed choice and response time patterns (Fig. 3).

### HDDMrlRegressor

The module described above cannot estimate the impact of continuous variables onto decision and learning parameters. The HDDMRegressor module was designed for this purpose for the DDM. We build on it here to introduce HDDMrlRegressor to estimate the impact of trial-by-trial regressors, such as time-varying data from brain imaging, onto learning and decision parameters from the RLDDM. As an illustration of its usage we next create and run a model estimating the impact of a trial-by-trial regressor onto decision threshold *a* (any parameter or combination of parameters is also possible). For this example, we assume a dataframe with a column “neural,” where the generated data on which the trial-by-trial regressor was sampled from a normal distribution with mean 0 and standard deviation of 1. For simulating choice and response data with the RLDDM a coefficient, with a fixed value of 0.2, was multiplied with the trial-by-trial regressor and added to the baseline decision threshold parameter, which was set to 1.

**Table:** 

m_reg = hddm.HDDMrlRegressor (data, ‘a ~ neural’, include=‘alpha’)							
m_reg.sample (1000, burn=250)							
m_reg.print stats ()							
	mean	std	2.5q	25q	50q	75q	97.5q
a_Intercept	0.994	0.006	0.984	0.990	0.993	0.999	1.008
a_neural	0.201	0.005	0.190	0.198	0.201	0.204	0.213

The code below illustrates how to create a model with continuous regressors and also shows that the model is capable of recovering the neural coefficient used for generating the data:

The columns in the table represent the mean, standard deviation, and quantiles of the approximated posterior distribution of each parameter. HDDM uses Patsy to create a linear model that estimates intercept and regressor parameters according to the model specification. More details about model specification can be found in the online tutorials for HDDM and the RLDDM modules in HDDM (http://ski.clps.brown.edu/hddm_docs/) and on the documentation for Patsy (patsy.readthedocs.org). Importantly, in contrast to HDDMrl, the HDDMrlRegressor module allows the user to estimate effects within participants while still ensuring proper updating of expected values. The model can further be used to estimate the effect of multiple regressors on multiple parameters (e.g., [’a ~ brain’,’t ~ eeg’]). Note that for the HDDMrlRegressor the learning rate parameter has to be specifically included (include=’alpha’), and that, as is the case also for the HDDMrl and Hrl modules, the estimated learning rate is not bound between 0 and 1, because it was transformed to improve sampling. To transform alpha back to the range 0–1 one can apply the inverse logit: *e*^alpha^/(1 + *e*^alpha^).

### Additional Functionality

#### RL with Softmax

HDDM also includes a traditional RL model that describes choice without RTs using the softmax choice function where the inverse temperature parameter describes propensity to explore vs exploit reward expectations. This model can be called with hddm.Hrl().

#### Separate learning rates for positive and negative RPE

Reinforcement learning models often fit data better if the learning rate parameter is allowed to vary by the sign of the prediction error (Frank et al. 2007; Gershman 2015; Niv et al. 2012). The HDDMrl and Hrl modules allow estimation of separate learning rates for positive and negative prediction errors by setting dual = True when defining the model, e.g., hddm.HDDMrl(data, dual = True).

### Parameter Recovery

An important validation step for a computational model is to determine how well its parameters are identified, particularly if the parameter estimates are interpreted or compared across groups. We generated 81 datasets with all combinations of three plausible values for each parameter (*a*: 1.5, 2.0, 2,5; *t*: 0,3, 0.4, 0.5; *α*: 0.15, 0.3, 0.45; *v*: 1.5, 2.25, 3.0). Within datasets, the parameter values assigned to subjects were drawn from normal distributions with the aforementioned values as the mean and the following standard deviations (*a*: 0.1, *t*: 0.02, *α*: 0.1, *v*: 0.25). Each dataset consisted of 40 synthetic agents performing 60 trials in each of the three conditions in the learning phase of probabilistic selection task. Example code for generating data can be found at (github.com/madslupe/hddm/blob/conflict_rlddm/scripts/param_recovery_rlddm.ipynb).

**Fig. 4:**
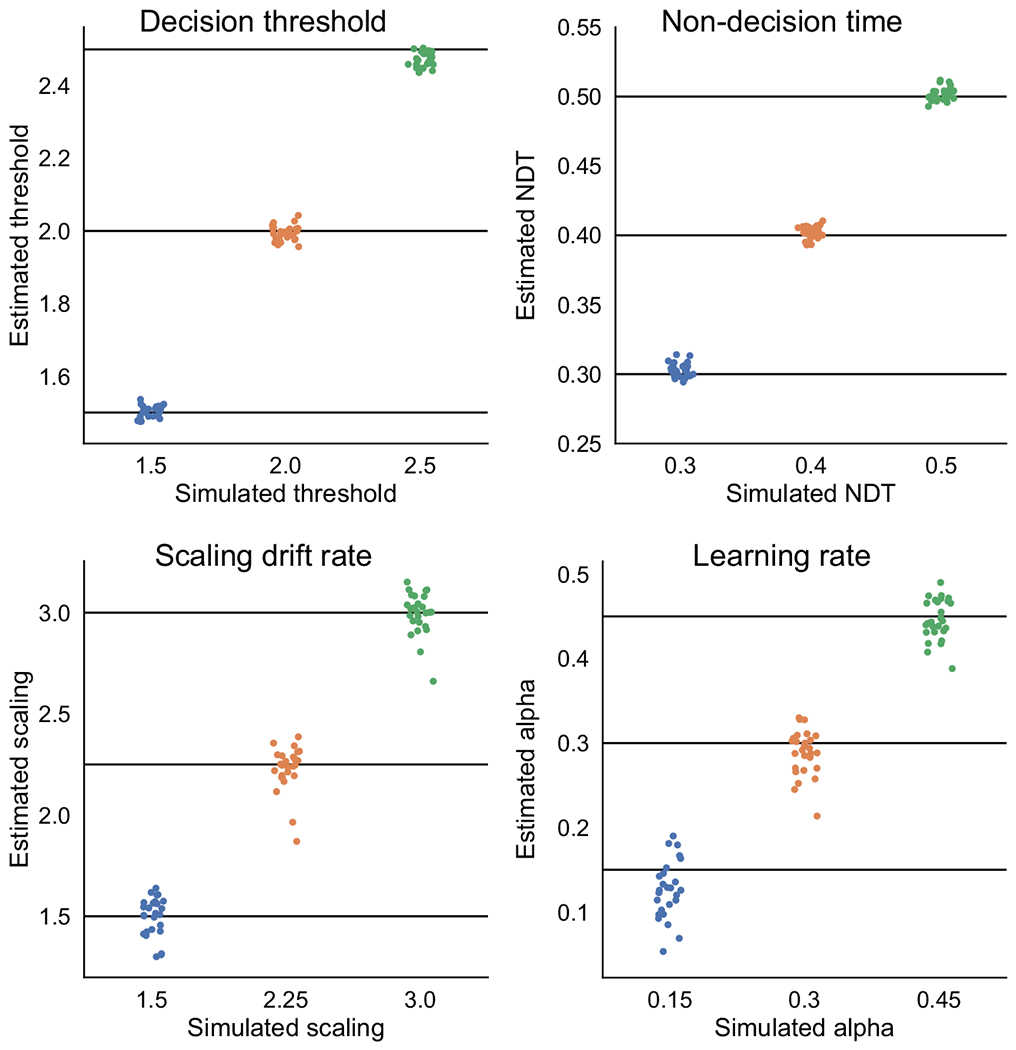
Parameter recovery. Simulated values on the *x*-axis and mean recovered group parameter values on the *y*-axis for the parameters decision threshold, non-decision time (NDT), scaling drift rate, and learning rate. Horizontal lines depict the true simulated values

Figure 4 shows the model’s ability to recover generated group parameters. The decision threshold and non-decision time parameters have excellent recovery, in that the estimated group parameters are strongly centered around the true mean, represented by horizontal lines. For the learning rate and scaling drift rate parameters, recovery is also adequate but somewhat more variable, likely related to the analogous collinearity between learning rate and choice sensitivity/inverse temperatrue in RL (Daw 2011). Indeed, the learning rate enters into the choice function indirectly by modulating the value scaled by the scaling factor.

### Parameter Recovery by Sample Size

**Fig. 5:**
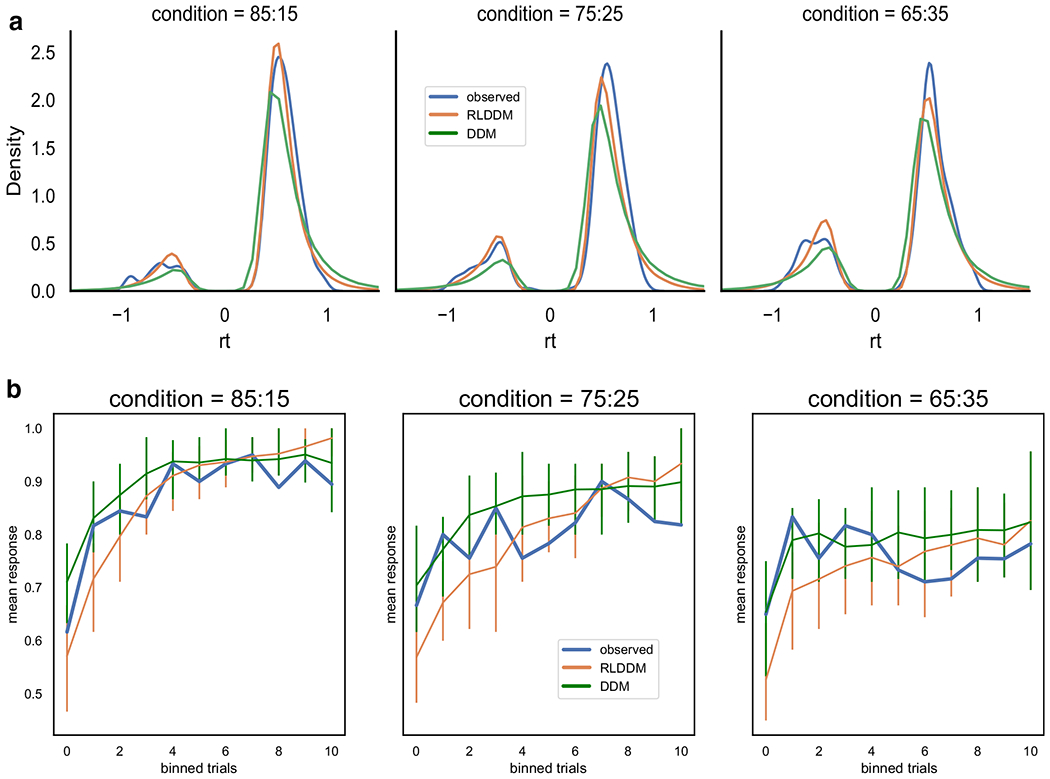
Absolute error for estimated parameters. The figure displays the absolute difference between the true generated parameter values and the mean estimated group parameter values across synthetic dataset with varying numbers of synthetic subjects and trials

An important concern for data collection is deciding on how much data to collect, in terms of both the number of participants to recruit and the number of trials to use to get reliable effects. To provide a guide to how reliable the model is in terms of recovering parameters, we generated data with different number of trials per subject while varying the number of synthetic subjects. Figure 5 shows the absolute error (the difference between simulated and estimated parameter values) for group parameters as a function of trials per condition and subjects in the learning phase of the PST. Linear regressions using error as the dependent variable revealed that error was reduced with increasing number of subjects (all *p* < 0.01) and trials (all *p* < 0.05), indicating that both more subjects and more trials for each subject should improve identifiability of parameters. There was a positive interaction effect of subjects and trials for all parameters, suggesting diminishing returns of increasing both, but this effect was only significant for alpha (*p* < 0.01).

### Mapping Neural Data onto Decision Parameters

We now turn to an illustration of how the newly developed model can be used to test how neural activation influences learning and decision making. In a recent study, Frank et al. (2015) tested predictions from a neural network model of the frontal cortex and basal ganglia in value-based decision making (Frank 2006; Ratcliff and Frank 2012). In this neural network model, the effective decision threshold is proactively raised when the model experiences decision conflict (when incompatible responses are coactive), via a pathway from mediofrontal cortex to the subthalamic nucleus (STN). While various studies have provided evidence for this notion using intracranial recordings within the STN, and/or deep brain stimulation of the STN, in patients with Parkinson’s disease (Cavanagh et al. 2011; Frank et al. 2007; Zavala et al., 2014; Herz et al. 2016b), Frank et al. (2015) assessed whether this function could be observed on a trial-to-trial basis in healthy participants, by capitalizing on variation in STN activity.

Participants performed a reinforcement learning task where the probability of reward differed across conditions and options, while their brain activity was measured through simultaneous electroencephalography (EEG) and functional magnetic resonance imaging (fMRI). When using HDDM to model decision making, neural regressors from the mediofrontal cortex (using both fMRI and EEG) were shown to interact with neural regressors from the STN to increase decision thresholds, particularly when learned reward values of alternative options were most similar (conflict). However, at this stage we had not developed the RLDDM to simultaneously estimate RL learning rates and DDM parameters, and hence rather than fit the learning rates, that study assumed that the expected rewards of options obeyed an ideal observer updating expectations according to Bayes’ rule (Frank et al. 2015). The trial-by-trial expectations from the ideal observer were then used to assess the degree of expected response conflict and were used as regressors, along with neural measures from the mediofrontal cortex and STN into a DDM.

Here we tested whether RLDDM would be able to recover similar effects and also estimate individual differences in learning parameters. We reasoned that in principle, the effects could get stronger, if the subject-specific estimates of learning rates improve the estimate of the conflict time series. However, it was also possible that some of the effects would no longer hold or would be better explained by other factors. We thus used the same dataset and the same regressors, but instead of taking expected values from an ideal observer we use the RLDDM to simultaneously estimate individual learning rates, decision parameters, and the influence of neural regressors onto parameters. Specifically, trial-by-trial values of decision threshold *a* were modeled as

(6) $$a_{i}∼a_{\text{Intercept}}+\text{stn*theta*conflict}+\text{presma*conflict},$$

which assumes an intercept for *a*, main-effects, and hypothesis-driven two-way interactions and one three-way interaction. STN is the blood-oxygen-level-dependent (BOLD) activity of the subthalamic nucleus, theta is the stimulus-locked theta-band power burst over mid-frontal sites as measured by EEG, pre-SMA is the BOLD activity in the pre-supplementary motor area (the neural locus of theta in that study), and conflict is the inverse of the difference in expected rewards between options (i.e., when options had similar reward values that constituted a higher conflict trial).

Trial-by-trial drift rates were calculated as

(7) $$v_{i}\sim Q\Delta *\left(v_{\text{Intercept}}+\text{caudate}\right),$$

which assumes trial-by-trial differences in expected values (*Q*Δ) are modulated by an intercept scaling parameter *v* and a slope-coefficient estimating the impact of trial-by-trial activity in caudate, following the assumption that activation in caudate could relate to reward expectations (Jocham et al. 2011), which could be accumulated (Ratcliff and Frank 2012; Ding and Gold 2010). We modified the HDDMrl model to estimate the effects and interactions of the neural regressors used in Frank et al. (2015). Although HDDMrlRegressor could be used to estimate neural regressor effects onto parameters, this module is (currently) not designed to estimate the effect of regressors onto inferred latent states, such as the conflict term used here, given that conflict itself depends on the learned values and hence learning rate. Code for allowing this function in the modified model can be found online (https://github.com/madslupe/hddm/tree/conflict_rlddm).

**Fig. 6:**
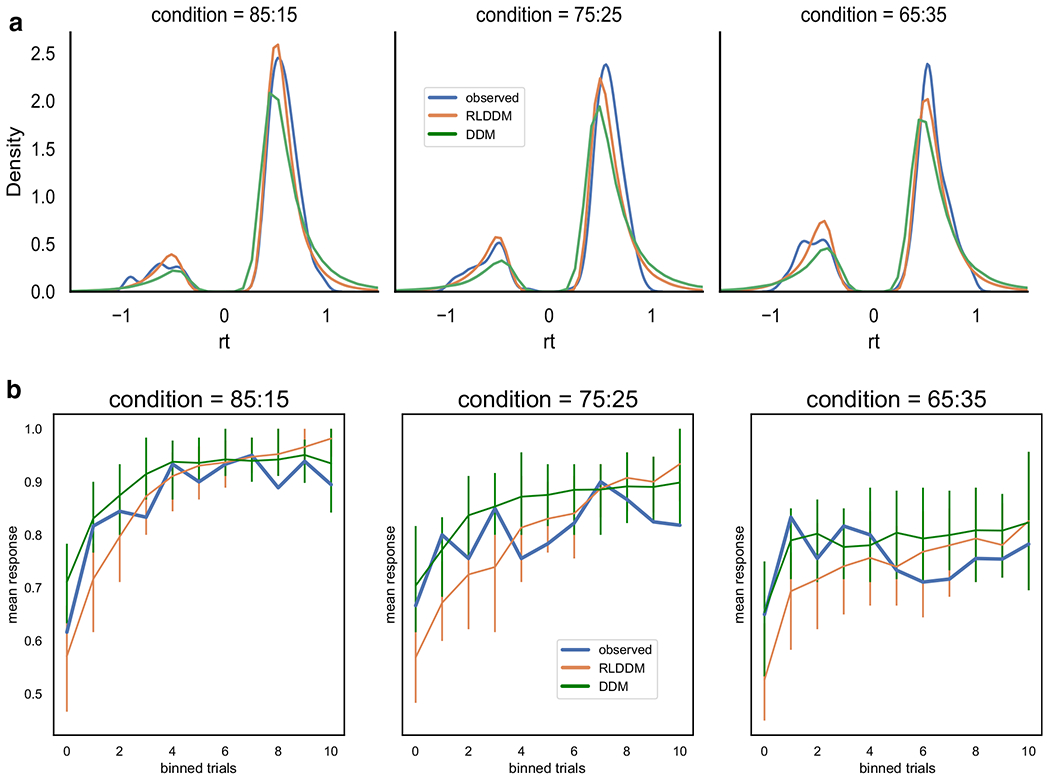
Posterior predictive checks. **a** Observed (blue) and predicted response time distributions across conditions from the RLDDM described here (orange) and the DDM model reported in Frank et al. (2015, green). RTs for lower boundary choices (i.e., worst option choices) are set to be negative (0-RT) to be able to separate upper and lower bound responses. Here, the models generally capture the response time distribution, except for predicting somewhat longer tails for choices in favor of the best option (i.e., positive RTs). The difference between the predictions of the models is best captured by the RLDDM being closer to the peak of the RT distributions for the two most difficult conditions. **b** Evolution of observed and predicted choice responses across difficulty conditions. The RLDDM underpredicts performance for initial learning, while the DDM overpredicts performance late in learning, in particular for the two most difficult conditions (75:25 and 65:35)

**Fig. 7:**
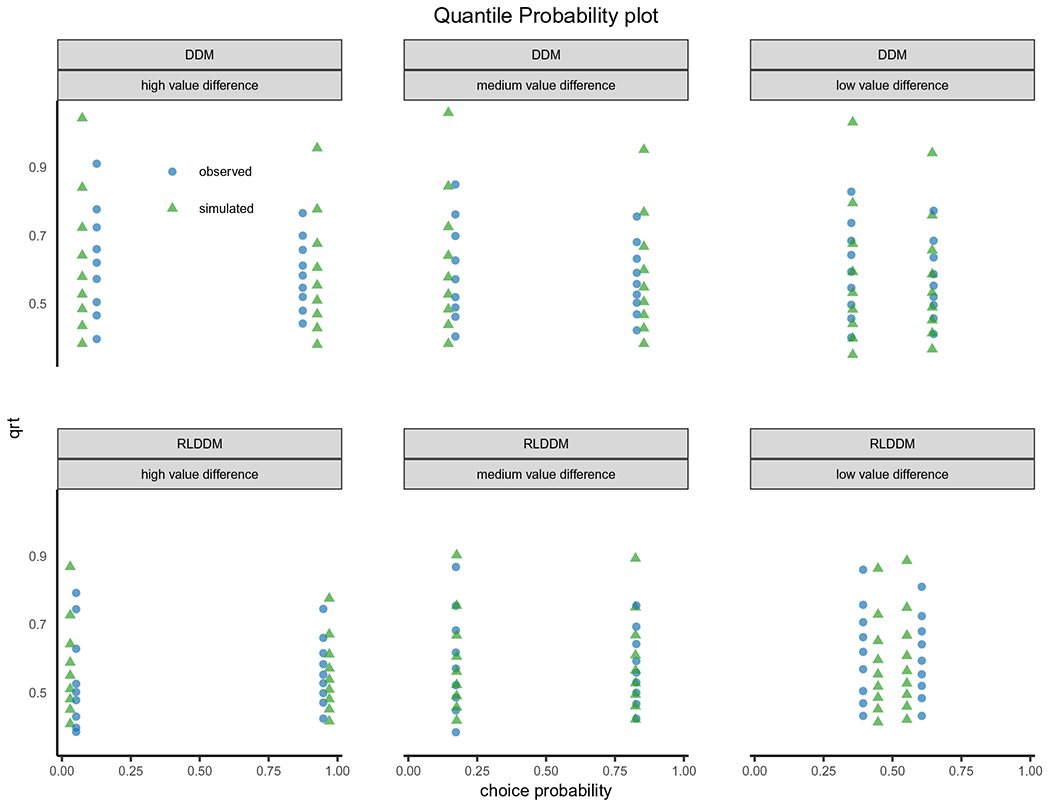
Quantile probability plots for the DDM (top) model reported in (Frank et al. 2015) and the RLDDM (bottom) model described here. The plot displays the observed (blue) and simulated (green) response proportion and RT quantiles for high-value (right-hand side of 0.5) and low-value (left-hand side of 0.5) choices across inferred levels of difficulty, using the difference in the mean of the beta learners for the DDM analysis and the difference in *Q* values for the RLDDM analysis, categorized into low, medium, and high differences in expected values. The plot shows that the DDM generally slightly overpredicts performance and predicts a longer tail of the RT distribution than observed. The RLDDM underpredicts performance for trials with an inferred low difference between estimated values but provides a better prediction of the observed RT distribution

The model fit, as measured by the deviance information criterion (DIC; Spiegelhalter et al. 2002), indicated that the RLDDM provided a better fit to the data compared to the previously reported model (DIC_rlddm_ = −357, DIC_ddm_ = 71). The Gelman–Rubin convergence statistic was below 1.1 for all parameters in both models. Posterior predictive checks on RT distribution (Fig. 6a) and choice evolution (Fig. 6b) across conditions indicated that both models captured the data, although the RLDDM seemed to better capture the RT distributions. In contrast, the RLDDM slightly underpredicted performance early on in the medium (75:25) and difficult (65:35) conditions. An alternative posterior predictive check is represented in Fig. 7. This quantile-probability (QP) plot (for more detail on QP-plots see, e.g., Ratcliff and McKoon 2008) shows the observed and predicted RT quantiles and choice proportions across inferred difficulty levels. These plots reiterate the results from Fig. 6, showing that the RLDDM better captures the RT distribution, while also showing that the RLDDM underpredicts performance when the difference in *Q* values is assumed to be low, i.e., when difficulty is high. See the “Discussion” section for potential ways to improve on this misprediction.

The RLDDM analysis reproduced the main conclusions from Frank et al. (2015), namely, that STN was associated with heightened threshold and that this effect was magnified when both midfrontal theta and conflict increased. Interestingly, these measures of significance, as calculated by the proportion of the posterior distribution of the regressor coefficient above zero, were numerically stronger in the RLDDM analysis (p(STN > 0) = 0.98, p(theta:stn:conflict > 0) = 1) compared to that reported in Frank et al. (2015) (p(STN > 0) = 0.96, p(theta:STN:conflict > 0) = 0.95), suggesting that the RLDDM, through improved model fit, might have increased the sensitivity to the influence of STN and midfrontal theta onto decision threshold, particularly as the latent “conflict” variable was now informed by the participant learning rates driving the expected values. An alternative account is that the learning is better described by the delta learning rule than an ideal observer (as was done in the original work), but in a robustness analysis, Frank et al. (2015) had also modeled learning with the delta learning rule (to obtain expected *Q* values before fitting the DDM, i.e., not simultaneously) and did not find effects as strong as reported here.

## Discussion

We have introduced modules that allow users to easily analyze instrumental learning data with the reinforcement learning drift diffusion model (RLDDM), which describes choosing in reinforcement learning using the drift diffusion model. The modules are built in the Python-based HDDM toolbox and have functions to estimate, assess, and validate models, in addition to estimating the impact of neural regressors onto learning and decision parameters. The validity of the model was illustrated by reanalyzing data showing that simultaneous estimation of learning and decision parameters increased sensitivity to how a mediofrontal-subthalamic network is related to decision threshold adjustments under conflict.

Bridging the fields of cognitive neuroscience and mathematical psychology in understanding how neural activation relate to cognitive processes has become increasingly popular in recent years (Turner et al. 2019). The approach described here allows users to directly test the association of neural data to latent variables of behavior. The models can further be used to relate dysfunction in cognitive or motivational processes to alterations in neural activation (Maia and Frank 2011).

During recent years several other hierarchical Bayesian toolboxes have been developed with the specific aim of simplifying the process of analyzing data with cognitive computational models. Popular toolboxes in R include the hBayes package (Ahn et al. 2017) that allows users to analyze data with the DDM and several RL models and the ggdmc package that includes sequential sampling models (Lin and Strickland 2020). The VBA toolbox in matlab also includes several RL models (Daunizeau et al. 2014). To our knowledge, the HDDM toolbox is the only that has functions to analyze data with the RLDDM and that facilitates estimating the impact of continuous regressors onto learning and decision parameters. However, custom models can be built in probabilistic programming languages with likelihood distributions for sequential sampling models, including Stan (Gelman et al. 2015) and JAGS (Plummer 2004).

### Limitations and Future Directions

Although the models described here offer flexibility in how to analyze data, given that they use the DDM choice function, they are currently restricted to two-alternative decision making tasks. Recent work (McDougle and Collins 2019) have extended reinforcement learning models that incorporate sequential sampling models with multi-alternative options using the linear ballistic accumulator (Brown and Heathcote 2008), but no such toolbox exists and incorporating this approach is beyond the scope of this paper.

The HDDMrlRegressor model allows users to map the effect of neural regressors onto decision and learning parameters, but it does not yet include a straightforward approach to map the effect of neural regressors onto inferred latent states, such as conflict measured by the inverse of difference in expected values. To capture the influence of the mediofrontal-subthalamic network on conflict described above, we modified the code of the HDDMrl model to incorporate the neural regressors. Future versions of the toolbox will hopefully include this function in a more general way, but until then, users can adapt the code to their needs using our modified code as an example (https://github.com/madslupe/hddm/tree/conflict_rlddm).

The choice evolution plot (Fig. 6b) showed that the RLDDM underpredicts initial learning while the quantile probability plot (Fig. 6c) revealed that the RLDDM underpredicted performance when the model inferred high difficulty, i.e., a small difference between expected values. These problems with prediction could be mitigated in multiple ways. As these choices were mainly from the initial trials, when expected values have not been sufficiently updated, a dynamic learning rate which scales with uncertainty could improve model fit (e.g., Behrens et al. 2007; Franklin and Frank 2015; Nassar et al. 2010). Alternatively, as shown by Fontanesi et al. (2019), a sigmoid transformation of the difference in expected values onto drift rate could capture the seemingly non-linear effect of expected values onto drift rate. Both of these alternatives could be added to the models in future versions of the toolbox.
